# Explanation

**Published:** 1852-07

**Authors:** 


### THE MEDICAL EXAMINER.

### PHILADELPHIA, JULY, 1852.

### EXPLANATION.

We have been concerned to learn, that some expressions, which occur in a communication from Dr. E. B. Gardette, of this city, published in our last number, have been the subject of misconstruction. A letter has been addressed to us by a gentleman from Baltimore, in which exception is taken to allusions indulged in by Dr. Gardette in the communication in question; inreference to hospitalities tendered him, on his visit to that city to attend the examination of the Dental College. That these allusions should have given offence to the gentleman who addresses us, we most sincerely regret. The language complained of, did not certainly strike us, on going through the press, as susceptible of an offensive interpretation. We must admit, however, that there are grounds for a call for explanation as to the allusions. We beg to express unreservedly our own regret that anything, appearing in the columns of this journal, should have pained the feelings of a highly respected correspondent from Baltimore. And we are authorized to add, on the part of Dr. Gardette, a no less unqualified expression of regret in the matter; and to disclaim, most pointedly in his behalf, any intention of attributing a sinister or unworthy design to the Faculty of the Dental College of Baltimore in their personal attentions to him, on his visit to that city.

